# Burnout syndrome and weekly workload of on-call physicians: cross-sectional study

**DOI:** 10.1590/S1516-31802012000500003

**Published:** 2012-11-13

**Authors:** Fabiano Timbó Barbosa, Bruna Acioly Leão, Gisélia Maria Sales Tavares, João Gustavo Rocha Peixoto dos Santos

**Affiliations:** I MSc. Professor in the Department of Clinical Surgery and Anesthesiology, Universidade Federal de Alagoas (UFAL), Maceió, Alagoas, Brazil.; II Medical Student, Universidade Federal de Alagoas (UFAL), Maceió, Alagoas, Brazil.

**Keywords:** Burnout, professional, Workload, Intensive care units, Cross-sectional studies, Diagnosis, Esgotamento profissional, Carga de trabalho, Unidades de terapia intensiva, Estudos transversais, Diagnóstico

## Abstract

**CONTEXT AND OBJECTIVES:**

Burnout syndrome (BS) is characterized by three dimensions: emotional exhaustion, depersonalization and reduced personal fulfillment. The objectives of this study were to evaluate a possible association between BS and weekly workload, and to describe the prevalence of BS and the sociodemographic and occupational profile of on-call physicians in Maceió.

**DESIGN AND SETTING:**

Cross-sectional study in intensive care units (ICU) at public and private hospitals in Maceió.

**METHODS:**

A self-administered form was used to evaluate sociodemographic characteristics and BS through the Maslach Burnout Inventory (MBI) among 67 on-call physicians at ICUs in Maceió. Pearson’s R correlation test was used to compare workload and emotional exhaustion. For other dimensions, Spearman’s S test was used (P < 0.05). Other variables were represented by simple frequencies. The 95% confidence interval was calculated for each variable.

**RESULTS:**

Among the physicians studied, 55.22% were female and the mean age was 43.9 ± 8.95 years. The mean weekly workload on call was 43.85 ± 24.49 hours. The frequency of high scores in at least one of the three dimensions of MBI was 70.14%.

**CONCLUSIONS:**

Despite the high prevalence of BS, especially among physicians who did not practice regular physical activity, our data did not indicate any significant correlation between weekly workload and any of the three dimensions of BS in this sample. The high prevalence of BS draws attention to the importance of investigating other possible causes, in order to prevent and adequately treat it.

## RESUMO

**CONTEXTO E OBJETIVOS:**

A síndrome de *burnout* (SB) caracteriza-se por três dimensões: exaustão emocional, despersonalização e redução da realização pessoal. Os objetivos deste estudo foram avaliar uma possível associação entre SB e carga horária semanal de trabalho, descrever a prevalência de SB e o perfil sócio-demográfico e ocupacional em médicos plantonistas de Maceió.

**TIPO DE ESTUDO E LOCAL:**

Estudo transversal em unidades de terapia intensiva (UTI) de hospitais públicos e privados de Maceió.

**MÉTODOS:**

Um formulário auto-aplicável foi usado para avaliar características sócio-demográficas e SB através do Maslach Burnout Inventory (MBI) com 67 médicos plantonistas de UTIs de Maceió. Foi realizado teste de correlação R de Pearson entre carga horária e exaustão emocional e, para as demais dimensões, foi utilizado o teste S de Spearman (P < 0,05). Demais variáveis foram representadas por frequência simples. Intervalo de confiança de 95% foi calculado para cada variável.

**RESULTADOS:**

Dos médicos estudados, 55,22% eram do sexo feminino, com média de idade de 43,9 ± 8,95 anos. A média da carga horária semanal de plantão foi de 43,85 ± 24,49 horas. A frequência de escores altos em pelo menos uma das dimensões do MBI foi de 70,14%.

**CONCLUSÕES:**

Apesar da alta prevalência de SB, especialmente nos médicos que não praticam atividade física regular, nossos dados não indicaram qualquer correlação significativa entre carga horária e qualquer uma das dimensões da SB na amostra. A alta prevalência da SB chama atenção para a importância da pesquisa de outras possíveis causas, para a prevenir e tratar adequadamente.

## INTRODUCTION

Burnout syndrome (BS) is a well-defined condition characterized by emotional exhaustion, depersonalization and reduced personal fulfillment.^1^ Emotional exhaustion consists of depletion of an individual’s emotional resources.^2^ It is considered to be the first feature of the syndrome and mainly occurs because of overload and personal conflicts in interpersonal relationships.^3^ Depersonalization is characterized by emotional numbness among professionals, who start to treat clients and colleagues as objects.^4^ This is a fundamental aspect of BS, given that the other features of BS can be found in depressive disorders in general.^2^ Finally, reduced personal fulfillment (or feelings of incompetence) reveals negative self-evaluation associated with unhappiness and dissatisfaction with work.^4^

Regarding the working environment of physicians in particular, some stressors that would raise the possibility of occurrence of BS can be highlighted: excessive demands that diminish the quality of care; long working hours; large numbers of shifts; low pay; requirement to deal with suffering and death; and constant exposure to risk, among others. In addition, the huge demands from society need to be taken into account. Society expects physicians to be infallible professionals, thereby creating pressure on them that is sometimes unsustainable.^3^

The reported prevalence of BS has varied widely among studies, depending on the population assessed and the conceptual values used as the reference point. The prevalence of BS in a study among intensive care physicians in Salvador, Bahia, was 63.3%, when taking into account high scores in at least one dimension. Thus, it was shown that there was high prevalence of the syndrome among the attending physicians studied.^4^

## OBJECTIVE

The objective of the present study was to evaluate a possible association between BS and the weekly workload of on-call physicians in six hospitals in Maceió, and to describe the sociodemographic and occupational profile and the prevalence of BS among those physicians. The hypothesis tested was that the correlation between BS and weekly workload among the on-call physicians in Maceió would be 0.3.

## METHODS

This was a cross-sectional observational descriptive study. The data was gathered from public and private intensive care units in Maceió, Alagoas, between January and February 2011.

The research subjects included were medical graduates who were working on call regularly and were registered with the Regional Medical Council of Alagoas. Subjects were excluded from the study if they did not provide complete responses to the Maslach Burnout Inventory (MBI), on the data extraction form, and if they did not fill out the question referring to their weekly workload. The study did not make any exclusions or distinctions in relation to age groups, gender, race or social group, based on the sample size calculation. Sixty-seven on-call physicians who met the inclusion criteria and who filled out the data extraction form completely were evaluated.

The participants selected were on-call physicians who were working within this type of regimen during the study period. We obtained data about their lifestyles, work, health and stress as well as any presence of BS among these doctors (as diagnosed by the MBI). On-call physicians were chosen because of the high stress burden that is associated with shiftwork, as well as with medical work in general, due to constant involvement in suffering and death, excessive workloads, low pay and large numbers of shifts, among other factors.^3^ This study was approved by the Research Ethics Committee of the Federal University of Alagoas (Universidade Federal de Alagoas, UFAL) before data-gathering started.

A consent statement was prepared for carrying out this project, since it was an investigation that dealt directly with people who voluntarily provided personal information, in response to a research protocol. The informed consent statement was presented to the subjects by the researchers prior to handing over the data extraction form, so that these individuals could be aware of all the issues involved in the investigation and could be assured of the security and confidentiality of their information. The participants were asked to fill out the form after firstly signing a consent statement. This document assured participants that the data gathered would remain confidential, assured them of the right to refuse to participate, explained that no risks were involved and enumerated the benefits from the research. This statement was developed in accordance with the model of the Research Ethics Committee of UFAL, and also in accordance with Resolution 196/96 of the Brazilian National Health Council and with the ethical principles stated in the World Medical Association’s Helsinki Declaration.^5^

Visits were made to two public and four private hospitals in Maceió, where physicians working in shifts were met, in order to invite them to participate. The information used was what the physicians themselves supplied on the data extraction form. Since the form was designed to be self-administered, the researchers did not interfere with the way in which the subjects filled it out. The forms were collected immediately after completion, and thus, there were only three forms that were not answered completely. The researchers continued to seek participants until the necessary sample size (calculated as described below) had been attained.

The data-gathering form consisted of four sets of questions: first set: general identification of the participants, with optional identification by name, with the aim of characterizing these individuals according to sex, marital status, age, expertise, length of employment, total weekly workload, work shifts, lifestyle, etc.; second set: evaluation of the level of burnout through the MBI; third set: questions about health problems, diseases and symptoms, in order to assess the overall health situation of the study population; fourth set: questions relating to stress in the workplace. The evaluation of symptoms and variables concerning personal, social, work and organizational characteristics correlated with the main symptoms presented in the BS process and its triggering variables.^6^

The MBI is the most commonly used questionnaire for assessing BS in studies on this syndrome worldwide. It was first published by Maslach and Jackson in 1978 and was translated into and validated for Portuguese by Tamayo in 1997. It is a questionnaire with 22 questions with five response options (Likert scale of 1 to 5) that address the three fundamental aspects of BS.^6^ Thus, each of the dimensions that characterize the syndrome was described independently. Emotional exhaustion was evaluated in nine items, depersonalization in five and personal fulfillment in eight. Cutoff scores in accordance with Tamayo’s adaptation were used for the five-point scale.^7^ For emotional exhaustion, scores greater than or equal to 27 indicated high levels; 19 to 26, moderate levels; and less than 19, low levels. For depersonalization, scores equal to or greater than 10 indicated high levels; 6 to 9, moderate levels; and below 6, low levels. The scores relating to personal achievement went in the opposite direction to the others, such that scores of zero to 33 indicated high levels; 34 to 39, moderate levels; and greater than or equal to 40, low levels.^7^ After enough forms had been filled out and collected at the end of the study, we analyzed the variables listed below.

The primary variables evaluated were BS and weekly workload. BS was defined as described by Grunfeld, who determined that the presence of a high level of any of its features, as measured by the MBI, establishes the diagnosis of the syndrome.^8^ The mean weekly workload of on-call work was defined as the level of greatest concentration of the number of work hours that an employee has an obligation to fulfill, according to law or employment contract, within the one-week period.

The secondary variables included: symptoms, signs and disorders associated with burnout syndrome, psychological and behavioral symptoms, medical specialty, length of time since graduating and starting professional work, type of establishment where the individual works, length of time working in intensive care, shifts, number of night shifts, length of time doing uninterrupted shifts, work elsewhere, monthly income, physical activity, leisure pursuits, smoking or alcohol habits, and chronic diseases.

The symptoms, signs and disorders associated with BS were characterized by progressive and constant fatigue, sleep disorders (sleep apnea, excessive sleepiness, insomnia or snoring), muscle or musculoskeletal pain, headaches, migraines, gastrointestinal disorders (intestinal flow difficulties, diarrhea or heartburn), immunodeficiency, cardiovascular disorders (high blood pressure), respiratory disorders and sexual dysfunction (such as impotence or loss of libido).^6^

The psychological and behavioral symptoms distinguished consisted of lack of attention and concentration, memory problems, slower thinking, feelings of alienation, feelings of loneliness, impatience, feelings of inadequacy, emotional instability, difficulty in self-acceptance, asthenia, depression, distrust, irritability, increased aggression, inability to relax, difficulty in accepting change, loss of initiative, increased substance use, tendency towards isolation, feelings of omnipotence, loss of interest in work or leisure, absenteeism, irony and cynicism.^6^

Among the factors within the working environment, the participants were asked to consider how stressful or harmful to their health the following were: excessive noise in the intensive care unit (ICU); the possibility of complications of inpatient care; administrative problems; dealing with suffering and death; obligation to deal with several issues simultaneously; number of patients per physician; pace of professional activities; lack of material resources; commitment of staff; relationship with the team; caring for terminally ill patients; pressure to discharge patients; and difficulty in sleeping when on night shifts.^9^

The following additional data were also analyzed: gender, age, marital status and presence of children.

It was calculated that 67 participants would be required, taking a correlation between MBI scores and the weekly workload of 0.3, a significance level of 5% and a statistical power of 80%. A virtual calculator was used, which was obtained through the following site: http://www.stattools.net/StatToolsIndex.php#Sample%20size.

The correlation between the emotional exhaustion score and the weekly workload was analyzed using Pearson’s R correlation test. Spearman’s S correlation test was used between the weekly workload and other dimensions. Both of these tests took a significance level of 5% and statistical power of 80%, and were two-tailed tests. The weekly workload was represented by mean ± standard deviation. The other variables were represented by calculating the simple frequency. The 95% confidence interval (CI) was used for each point that was estimated.

## RESULTS

Sixty-seven intensive care physicians were evaluated and included in the study, as envisaged in the sample size calculation. Among these physicians, 55.22% (37/67; 95% CI: 43.32 to 67.12) were female and the mean age was 43.9 ± 8.95 years (95% CI: 41.76 to 46.04). Regarding marital status, 58.2% (39/67; 95% CI: 46.39 to 70.01) were married and 20.89% (14/67; 95% CI: 11.16 to 30.62) were single, while the other 20.89% (14/67; 95% CI: 11.16 to 30.62) had other marital statuses (separated, divorced, widowed, etc.). Among the physicians studied, 77.61% (52/67; 95% CI: 67.63 to 87.59) had children.

With regard to specialist titles, 77.61% (52/67; 95% CI: 67.63 to 87.59) of the study population had a title, but 53.73% (36/67; 95% CI: 41.8 to 65.66) did not have a specific title in intensive care. The mean length of time since graduation was 19.4 ± 9.08 years (95% CI: 17.22 to 21.57), the mean length of time in professional work was 17.74 ± 8.95 years (95% CI: 15.6 to 19.89) and the mean length of time doing ICU work was 14.01 ± 8.53 years (95% CI: 11.95 to 16.07).

Regarding the type of establishment, 19.4% (13/67; 95% CI: 9.94 to 28.86) worked only in public institutions; 22.39% (15/67; 95% CI: 12.41 to 32.37) only in private institutions; and 58.2% (39/67; 95% CI: 46.39 to 70.01) in both types. Considering the type of ICU, 29.85% (20/67; 95% CI: 18.9 to 40.8) worked only in general ICUs; 4.48% (3/67; 95% CI: 0.47 to 9.43) only in cardiac ICUs; and 43.28% (29/67; 95% CI: 31.42 to 55.14) only in pediatric and/or neonatal ICUs. Another 22.38% (15/67; 95% CI: 12.4 to 32.36) also worked in other types of ICU (obstetric and neurologic, among others). Overall, 48.25% (33/67; 95% CI: 36.29 to 60.21) worked in general ICUs; 14.92% (10/67; 95% CI: 6.39 to 23.45) in cardiac ICUs; 52.24% (35/67; 95% CI: 40.28 to 64.2) in pediatric and/or neonatal ICUs; and 29.85% (20/67; 95% CI: 18.9 to 40.8) in others.

The average weekly workload on shifts among the physicians studied was 43.85 ± 24.49 hours (95% CI: 37.98 to 81.83). Of these, the average length of uninterrupted on-call work was 23.27 ± 10.91 hours (95% CI: 20.63 to 25.9). Considering the shifts, 64.18% (43/67; 95% CI: 52.7 to 75.66) of the physicians worked three shifts (morning, afternoon and evening); 22.38% (15/67; 95% CI: 12.4 to 32.36) some combination of two shifts; and 13.43% (9/67; 95% CI: 5.27 to 21.59) only worked one shift. The modal distribution of nightshifts was 2.

Out of the surveyed physicians, 88.05% (59/67; 95% CI: 80.29 to 95.81) worked over the weekend. The average workload over the weekend was 18.98 ± 9.14 hours (95% CI: 16.56 to 21.39). It was observed that 85.07% (57/67; 95% CI: 76.54 to 93.6) of the physicians worked in other places besides the ICU (outpatient clinic, emergency service and wards, among others), and the mode was at least one other work location for these physicians.

Among the physicians studied, 40.30% (27/67: 95% CI: 28.55 to 52.05) gave responses to the item on monthly income, which was an optional item. The respondent’s mean reported monthly salary was 14,233.33 ± 5,863.91 reais (95% CI: 12,021.49 to 16,445.17). Of these physicians, 37.04% (10/27; 95% CI: 18.82 to 55.25) received an amount less than or equal to R$ 10,000, while 62.96% (17/27; 95% CI: 44.74 to 81.17) had an income greater than R$ 10,000.

Physical activity was also addressed, and 58.21% (39/67; 95% CI: 46.40 to 70.02) of the physicians reported practicing physical activity on a regular basis, with a frequency of around 2-4 times a week for 89.74% of them (35/39; 95% CI: 80.22 to 99.26). The most frequently reported activities were weight training, aerobics, walking and running. It was observed that 50% (14/28; 95% CI: 31.48 to 68.52) of the participants who did not practice physical activity had a high level of emotional exhaustion.

With regard to general health conditions, corresponding to lifestyle and unhealthy addictions, 97.01% (65/67; 95% CI: 92.93 to 101.08) of the physicians interviewed said that they were nonsmokers. Alcohol consumption was reported by 53.73% (36/67; 95% CI: 37.44 to 70.02) of the sample. This was generally reported as social consumption, and the evaluation was hampered by subjectivism. In addition, 61.19% (41/67; 95% CI: 49.52 to 72.86) reported that they had chronic diseases, consisting mostly of hypertension, cardiovascular disorders and sleep disorders.

The characteristic physical symptoms of the syndrome are sleep disorders, headaches, constant fatigue, progressive muscle or musculoskeletal pain, headaches, respiratory disorders, gastrointestinal disorders, sexual dysfunction, immunodeficiency and cardiovascular disorders. In our study, the disorders most frequently reported by the physicians were: sleep disorders in 53.73% (36/67; 95% CI: 41.79 to 65.67); followed by muscle or musculoskeletal pain in 37.31% (25/67; 95% CI: 25.73 to 48.89); gastrointestinal disorders in 34.33% (23/67; 95% CI: 22.96 to 45.70); and progressive, constant fatigue in 28.36% (19/67; 95% CI: 17.57 to 39.15). Only 13.43% (9/67; 95% CI: 5.26 to 21.59) reported not having any of the disorders mentioned.

Factors within the workplace that contribute towards development of the syndrome, those which are perceived as harmful to health and lead to emotional exhaustion were also addressed. Thus, 77.61% (52/67; 95% CI: 67.63 to 87.59) of the physicians reported that administrative problems that they faced in the workplace, regardless of whether the workplace was public or private, interfered with their health and raised their stress levels, thereby leading to impatience and irritability. Lack of material resources was also reported as stressful by 64.18% (43/67; 95% CI: 52.70 to 75.66). The number of patients per physician was also mentioned as a source of stress by 52.24% (35/67; 95% CI: 40.28 to 64.20). The fast pace of professional activities was mentioned as a cause of stress by 43.38% (29/67; 95% CI: 31.42 to 55.14) and lack of commitment among the team was also mentioned by 43.38% (29/67; 95% CI: 31.42 to 55 14).

Among the psychological symptoms and behavioral and psychological characteristics of burnout in this population, 64.18% (43/67: 95% CI: 52.70 to 75.66) reported impatience, followed by 52.24% (35/67; 95% CI: 40.28 to 64.20) of the sample that also reported irritability. Memory impairment was reported by 37.31% (25/67; 95% CI: 25.73 to 48.89); feelings of discouragement by 35.82% (24/67; 95% CI: 24.34 to 47.30); slower thinking by 34.33% (23/67; 95% CI: 22, 96 to 45.70); and inability to relax by 31.34% (21/67; 95% CI: 20.23 to 42.45).

Among the research subjects, 34.32% (23/67; 95% CI: 22.96 to 45.70) felt unable to act in accordance with their principles at work, which may reflect the organizational characteristics of their establishments, regardless of whether public or private, which thus contributed towards development of the syndrome. Feelings of discomfort caused by frequent changes of rules and regulations at the workplace were also reported, and also the feeling that the quality of the workplace atmosphere affected production, by 20.89% of the physicians (14/67; 95% CI: 20.15 to 30.62).

The frequency of high scores in at least one of the three dimensions of MBI was 70.14% (47/67; 95% CI: 63.66 to 80.62). BS was diagnosed when high scores were observed in at least one of the three dimensions. The frequency of high scores in all three dimensions of MBI was 17.91% (12/67; 95% CI: 8.72 to 27.1) and the frequencies of high score in each of the three dimensions analyzed separately were: 41.79% (28/67; 95% CI: 29.88 to 53.6) for emotional exhaustion; 37.31% (25/67; 95% CI: 18.36 to 56.26) for depersonalization; and 58.2% (39/67; 95% CI: 46.39 to 70.01) for inefficiency.

Pearson’s R correlation test was used between emotional exhaustion and weekly workload, while Spearman’s S correlation test was used between the workload and the mean scores for personal fulfillment and depersonalization. There were no significant correlations for any of the dimensions of BS, in relation to the weekly workload (Table 1).

**Table 1.** Correlation between weekly workload and each of the three dimensions of burnout syndrome

Dimensions	r	P-value
Emotional exhaustion^1^	+ 0.053	0.6684
Depersonalization^2^	+ 0.067	0.5879
Personal fulfillment^2^	- 0.066	0.5946

r = correlation value. ^1^Pearson’s R correlation; ^2^Spearman’s S correlation.

## DISCUSSION

In this study, we sought to investigate whether any correlations existed between weekly workload and the three dimensions of BS, among intensive care physicians working in Maceió. However, no significant correlation was found in the studied sample.

While there are other instruments for measuring this syndrome, MBI is the one that is most used worldwide to independently describe each of the dimensions that characterize BS.^7^^,^^10^ There is still no consensus in the literature regarding the diagnosis of the syndrome. According to Ramirez et al., only individuals who exhibit the three characteristic dimensions of professional burnout can be considered to present the syndrome.^11^ However, we took the diagnosis of the syndrome to be in accordance with Grunfeld et al., who classified burnout as the presence of at least one high level, regardless of whether this consisted of emotional exhaustion, depersonalization or low levels of personal fulfillment.^8^ Thus, although the Maslach questionnaire is specific for diagnosing the syndrome, the lack of standardization in evaluating the results makes them difficult to interpret, which may explain our finding that large numbers of people presented BS. New studies are being performed to find instruments that better assess the Brazilian reality.^10^

In our research, we combined application of MBI with other factors of relevance for characterizing individuals and their working conditions. All the participants answered the MBI completely, but some items in the other sets of questions on the form were left unanswered or were not answered as expected, thus causing difficulty in statistical analysis and standardization. However, this did not hinder the objective of the study.

In cross-sectional studies, groups are formed only at the data analysis stage, as it is known at this point which individuals are exposed or not exposed to the variables, or which of them are healthy or sick. Cross-sectional studies examine the relationship between exposure and disease in a given population or sample, at a particular time, thus providing a picture of how the variables are correlated at that time. Therefore, this type of study does not establish causality and just points out the association between variables.^4^ The doctors answered the questions during their shifts, which may have compromised the responses, because of possible difficulty in fully concentrating on the form.

The profile of the critical care physicians studied consisted of a relatively young population, with slight female predominance. By analyzing burnout as a syndrome (rather than as independent variables), women health workers were seen to make up the most vulnerable group, and this can perhaps be explained by excessive workloads when summed their family commitments.^7^ The physicians surveyed had on average graduated more than 10 years earlier and also had done more than 10 years of ICU work, and a slight majority did not have a specialist title in intensive care. These findings contrast with what was observed in similar studies, such that the physicians’ mean age, length of time since graduation and length of work in ICUs were slightly greater in the present study.^4^^,^^9^^,^^12^ Having a specialist title would indicate a desire to spend more time working in the ICU, which would imply that physicians who possessed this title would have a lower frequency of BS.^4^

Most of the interviewees were married and had children: the literature indicates that people who are married or are living with a stable partner and who have children experience feelings of family responsibility and greater resistance to the syndrome, through an associated ability to face emotional problems.^7^

A study on oncologists found a connection between working exclusively in public institutions and lower levels of emotional exhaustion.^2^ It was suggested that for these professionals, it was rewarding to work with students as part of the work in hospitals and public institutions, and that these institutions were places that offered less bureaucracy and greater creative freedom for professionals.^2^ However, this rationale may not apply in relation to working in intensive care, particularly because of greater difficulties regarding working conditions, thus leading to greater emotional distress. Accordingly, 77.61% of the physicians interviewed reported administrative problems as stressors in the workplace.

The frequency of BS in this study, when taking into account a high score in at least one dimension, was 70.14% (47/67; 95% CI: 63.66 to 80.62). This level was higher than what was observed in other studies, using the same criteria.^2^^,^^4^^,^^9^^,^^12^ Emotional exhaustion was the dimension that had the second highest frequency in our study. It is regarded as the first reaction to the stress generated by the demands of work. Once exhausted, people feel physical and emotional fatigue, with difficulty in relaxing and performing their activities. The internal resources that professionals possess for facing up to situations experienced at work, as well as their energy available for performing these activities become reduced. The characteristics of this dimension, compared with the others, allow it to be easily accepted by professionals in expressing consistent aspects of burnout.^4^

Given the psychological and physical symptoms, professionals develop depersonalization, which is characterized by cold and negative attitudes, thereby generating derogatory treatment towards the people directly involved with the work.^12^ They even start to become cynical and ironic towards the recipients of their work.^9^ However, depersonalization was the least prevalent dimension in the subjects studied. This can possibly be explained by the difficulty that physicians feel in accepting some of the claims associated with this aspect of the syndrome, since these involve attitudes that are generally not well accepted by society.

If these professionals feel that they are inefficient, with diminished self-confidence and a sense of failure, there is a reduction in personal fulfillment at work.^4^ It is important to emphasize that this dimension is considered by some authors to be the last reaction to the stress generated by work demands.^4^^,^^8^^,^^9^^,^^12^ Nonetheless, this was the dimension with the greatest frequency of high levels among these research subjects, and it did not appear to be related to salaries, weekly workload or number of years of doing the work. It was beyond the objectives of the present study to investigate possible correlations with this finding, although it was noted that there were correlations between the syndrome scores and the weekly workload, as well as between these scores and the length of time working in the ICU. This would suggest that the greater the workload was, the lower the scores for personal fulfillment would be, and consequently, the higher the frequency of high levels of this dimension would be, which also happened with shorter lengths of ICU work in years. However, the significance of these correlations could not be proven in this study.

The greater the subjects’ ages and therefore the longer that they had been working, especially doing ICU work (which would indicate greater experience and appreciation of the field), the lower the frequency of burnout was, such that younger workers were more susceptible.^7^ This can be explained by the fact that at the beginning of the profession, young physicians are at a time of growing awareness of realities, in which the transition from idealistic expectations to the reality of everyday practice shows that the reality does not always turn out as promised or expected.^7^

Considering that BS affects about one in every two physicians, of whom one third are significantly affected and one tenth present a severe form, with irreversible characteristics, evaluation of the prevalence of this syndrome and its possible causes is of great importance.^2^ In particular, intensive care physicians suffer greater stress than seen in other specialties, due to greater exposure to death, which conflicts with the purpose of healing for which the doctor was trained.^4^ ICU work also continuously requires updates of knowledge and scientific qualifications, along with abilities, attention, quick thinking and emotional control in dealing with patients, family members and the team as a whole.^4^ All of these requirements may be compromised by this syndrome. The results from the present study, combined with other available studies, indicate that there is a need for further research on occupational stress, given the negative impact and effect on workers’ health and on hospital institutions.^13^ Physicians are directly connected to this reality by being exposed to extensive working hours, regardless of the type of service, and bear a heavy burden placed on them by society, through their role of extremely high responsibility.^3^ Therefore, practical efforts should be directed towards decreasing the stressors in the workplace that contribute towards developing the syndrome, which is perceived as harmful to health and leads to emotional exhaustion.

BS remains unknown among many health professionals, and greater clarification is required in relation to the syndrome, its manifestations and causes, and consequently in relation to effective ways for treating and preventing it, and for intervening in it. Clarification is also needed in order to understand the impact that the work has on the professionals’ lives and consequently on the quality of services provided for the population.^3^

This was a pioneering study in the sense of providing a profile of doctors working in ICUs in Maceió and assessing the frequency of BS in this population. However, the limitations mentioned above regarding the study method and its objectives need to be taken into consideration. This study raised the hypothesis of a correlation between high weekly workload and BS, which could not be proven in this population. However, from the study results, we can raise hypotheses about other sociodemographic and work-related factors that may be associated with BS among ICU physicians. For better confirmation of this result, statistical analysis of greater complexity would be required, with better organization of the data extraction form to obtain more objective variables and responses. A more detailed statistical analysis on other variables that could be associated with the syndrome was not part of the main objective of this study, but this needs to be addressed in future studies, with the purpose of clarifying the causal factors of BS.

The high prevalence of BS draws attention to the importance of assessing other possible causal factors, in order to adequately prevent and treat it, since it may affect the quality of the services offered by physicians and the success of medical interventions. Thus, it is recommended that future studies should attempt to ascertain correlations between BS and other relevant variables. Since reduced personal fulfillment was the most frequent dimension observed in the sample, this particular dimension may need further investigation, given that it indicates that people are unhappy with their work. It may also be important to reevaluate the use of the MBI for diagnosis, since it may not be the most adequate tool for Brazilian realities and its scores (especially for personal fulfillment) may lead to excessive diagnosis.

## CONCLUSION

Despite the high prevalence of BS, especially among physicians who did not practice regular physical activity, our data did not indicate any significant correlation between weekly workload and any of the three dimensions of BS among intensive care physicians working in Maceió.
